# Workers’ Healthcare Assistance Model (WHAM): Development, Validation, and Assessment of Sustainable Return on Investment (S-ROI)

**DOI:** 10.3390/ijerph17093143

**Published:** 2020-04-30

**Authors:** Lilian Monteiro Ferrari Viterbo, André Santana Costa, Diogo Guedes Vidal, Maria Alzira Pimenta Dinis

**Affiliations:** UFP Energy, Environment and Health Research Unit (FP-ENAS), University Fernando Pessoa, 4249-004 Porto, Portugal; decovirtual@yahoo.com.br (A.S.C.); diogoguedesvidal@hotmail.com (D.G.V.); madinis@ufp.edu.pt (M.A.P.D.)

**Keywords:** Workers’ Healthcare Assistance Model (WHAM), patient-centred care, integrated care, interdisciplinary, sustainable return on investment (S-ROI), economic sustainability, WELLCAST ROI™

## Abstract

The present study aimed to present and validate the Worker´s Healthcare Assistance Model (WHAM), which includes an interdisciplinary approach to health risk management in search of integral and integrated health, considering economic sustainability. Through the integration of distinct methodological strategies, WHAM was developed in the period from 2011 to 2018, in a workers’ occupational health centre in the oil industry in Bahia, Brazil. The study included a sample of 965 workers, 91.7% of which were men, with a mean age of 44.9 years (age ranged from 23 to 73 years). The Kendall rank correlation coefficient and hierarchical multiple regression analysis were used for the validation of WHAM. The assessment of sustainable return on investment (S-ROI) was made using the WELLCAST ROI™ decision support tool, covering workers with heart disease and diabetes. WHAM can be considered an innovative healthcare model, as there is no available comparative model. WHAM is considered robust, with 86% health risk explanatory capacity and with an 85.5% S-ROI. It can be concluded that WHAM is a model capable of enhancing the level of workers’ health in companies, reducing costs for employers and improving the quality of life within the organization.

## 1. Introduction

More than ever, life, as we know, will never be the same. The world is currently experiencing the coronavirus pandemic (COVID-19) [1], an unforeseeable health development that is affecting the entire global population, and consequently healthcare assistance models across the globe. There is now an urgent need to look at human health through the “one health” lens [2], to design and implement programs, policies, legislation, and research in a cooperative manner among all sectors of society to achieve better public health outcomes.

In addition to the recognition of the success of the current healthcare models in the relief of pain and the treatment of multiple pathologies, several criticisms are gaining support, pointing out the limitations relating to the attention to patient health. These issues include approaches that take an undifferentiated view of the individual, which is focused exclusively on the part of the body that is sick; the focus on the curative actions of diseases, injuries, and damages; the advancement of medicalization; and the generalization of hospital care using technology. In the past, if a medical doctor was seen as a figure possessing the knowledge necessary to cure the patient, nowadays that figure is seen as one part of a team, with the patient being the final decision-maker in their health outcomes.

The World Health Organization has chosen to strengthen people-centred care and integrated health services as priority strategies to transform health services to meet the health challenges of the 21st century [3]. This favours the emergence of integrated care models, which are seen as possible solutions to the growing demand for improvement in the patient experience, especially in patients with chronic conditions.

Considering economic sustainability in the search for integral and integrated health, this study aims to present and validate a model of workers’ healthcare, the Workers´ Healthcare Assistance Model (WHAM), which embraces an interdisciplinary approach towards health risk management.

In light of the literature review, the following three research hypotheses were formulated:

**Hypotheses** **(H1).** *WHAM promotes integral and integrated care;*

**Hypotheses** **(H2).** *WHAM is robust and has greater explanatory capacity for workers’ health risks;*

**Hypotheses** **(H3).** *WHAM is economically sustainable and provides a significant return on investment.*

## 2. Literature Review

A review of the literature in the field of occupational health highlights discussions relating to “assistance models”, a term that varies based on the conceptualization, which can include “assistance modalities or technological models” [4,5]; “ways to promote health” [6]; “assistance models” [4,6,7]; “technical, techno-assistance, and technical assistance models” [4,8]; “modes of intervention” [7]; “attention models” [9,10,11]; and “care models”. The result of this diversity of terms is the already identified difficulty in conceptualizing assistance models. Healthcare assistance models are understood as technological combinations with different purposes, which are used to solve problems and meet needs within a given context and population and in a given territory (individuals, groups, or communities), to organize health services or to intervene in situations, depending on the epidemiological profile and investigation of health problems and risks [12]. These logical systems organize the functioning of care networks, articulating the relationships between network components and health interventions. In turn, these are defined according to the prevailing view of health, demographic and epidemiological situations, and social determinants of health at a given time and in a given society and place [13].

According to Campos [5,6], the conceptualization of an assistance model, technological model, or assistance modality must go beyond mere organizational and technical design, showing a new way of producing assistance actions anchored in the organization of the state.

According to Silva [14], biomedicine has become the hegemonic model in the provision of health services in Brazil and other countries around the world, influenced by accumulated knowledge and the paradigm of science. In this process, the daily requirements in the health sector stand out, such as the relationships between people; the involvement and co-responsibility of managers, health professionals, and patients in healthcare; as well as the bond, reception, and humanization of healthcare assistance practices [15]. From a technological point of view, there is a predominance of the use of the so-called “hard technologies” (equipment), to the detriment of light technologies (professional-patient relationships) [8,16]. Thus, diagnostic tests are a priority, but patients are not necessarily considered in terms of their suffering. This approach has been the target of criticism at the international level, starting from the 1970s and gaining greater importance in the second half of the 1980s [11,17]. In terms of the biomedical model, there is a certain neglect of the importance of the determinants of the health–disease process; that is, the focus on the disease and not on the elements that contribute to health promotion, underestimating that cultural, ethical, and social aspects condition lifestyles and that these are also determinants in the same process [13,14,18].

Merhy [8] contributes to the debate about the need to change the hegemonic assistance model, arguing that it is necessary to impact the core of care. In this sense, it is necessary to invest in relational-type light technologies, focusing on the needs of users and reversing the investment in hard or light-hard technologies, which can be translated into standards, equipment, and materials. Thus, light technologies are used and combined with people and resources to achieve certain objectives, which are gathered in an organized manner and consolidated as essential elements of health services [19].

Regardless of the scope, health services are always complex. The processes are standardized by regulatory bodies, service providers, and class representatives, among others. They have highly specialized and qualified workers who, belonging to different class councils, have interests that do not always converge [20]. Team composition characteristics in health services must be highlighted, recognizing these team members as the main actors responsible for the implementation of technologies aligned to a healthcare assistance model. Faria [21] draws attention to the fact that actions performed in a given place to deal with a certain problem may not apply to other situations, considering the historical-political context that influences a situation. Therefore, the use of healthcare assistance models invariably requires the selection of certain constructs that support them. Thus, they can be used in an alternative or adapted way, as long as they enable the achievement of similar results. To incorporate new health needs, healthcare assistance models can be considered to have influenced the organization of care models, being more focused on specific populations, such as the chronically ill. A comprehensive care model defines how health services are offered, providing the best care and service practices for a person or population group as they evolve through a condition, injury, or event, aiming for people to receive the right care, at the right time, by the right team, and in the right place [22].

The field of occupational health is a fertile environment for the development of interdisciplinary practices [23,24,25,26], as it encompasses knowledge from different disciplines, requiring constant and complex interactions between professionals in the fields of epidemiology, the environment, engineering, and healthcare, among others. The framing of occupational health in a biomedical healthcare assistance model favours the development of disjointed and ineffective interventions regarding the needs presented by workers, while the biopsychosocial model is often used in their work environments. According to Annadale [27], the biomedical healthcare assistance model only focuses on the physical processes, i.e., the pathology, biochemistry, and physiology of a disease, neglecting the roles of social factors or individual subjectivity.

In this context, it is necessary to discuss a model of assistance in occupational health that is capable of reviewing the central characteristics of the biomedical healthcare assistance model, including: (i) organization of practices focused on the identification of signs and symptoms and the treatment of diseases, with health promotion not being a priority; (ii) assistance is organized based on individual spontaneous demand, with an emphasis on specialization and the use of hard technologies; (iii) the work is developed in a fragmented, hierarchical manner and with inequality across different professional categories; (iv) difficulty in implementing the integrated care due to the lack of understanding of the individual as a multidimensional human being, as well as the lack of communication and integration between the services involved; (v) health planning is seldom used as a management tool; (vi) the training of health professionals is specialized, based on the hegemony of scientific knowledge; and (vii) themes such as interdisciplinary, people-centered care, attachment, and welcoming are not prioritized. Another aspect of great relevance in the current global context of scarcity of resources, particularly in the current context of COVID-19, is the prioritization of investments ineffective, integral, and integrated interventions, which can be achieved through a model that contemplates the management of occupational health risks, considering the social health determinants [28,29], global disease burden [30], environmental aspects [31,32], sustainable development goals [33,34] and in particular, working conditions that affect an individual’s health [35].

In the current context, the effectiveness of a healthcare assistance model must include economic sustainability in addition to health gains, to know how much the company has earned due to investments made in a certain area, with the sustainable return on investment (S-ROI) being a very important metric for this assessment. Measuring the S-ROI [36,37,38] of preventive programs is not an easy task, due to the large number of variables that influence this calculation. The main variable is patient health, which can improve or worsen unpredictably. Analyzing the S-ROI in preventive programs identifies the financial impact a program generates concerning the amount invested, which must be considered in the long term. Disease prevention actions bring future returns, mainly to the reduction of healthcare assistance costs. If the individual participates in preventive programs, the probability of developing diseases or discovering them in advanced stages decreases. Over the past 20 years, several studies [39,40,41,42,43,44,45,46,47] have addressed this issue and there is growing evidence that workplace programs can generate acceptable financial returns for employers investing in them. A study of Johnson and Johnson employees [39] showed a difference in the increase in the average annual costs of internment between workers involved and not involved in lifestyle improvement programs and changes in the workplace, representing $43 and $76, respectively, thus representing a considerable increase in percentage terms. The study by Munir et al. [45] aimed to conduct a cost-benefit analysis of the stand more at work (SMArT) workplace intervention, designed to reduce sitting time. A net saving of $2.18813 (95% CI; $−4.3804; $4.8143) per employee was found as a result of productivity increase. Peik and others [46] applied the Research and Development (RAND) Europe model, a program designed to expand access to up to 40 evidence-based clinical preventive services for all employees and eligible family members, as part of a unique global health initiative at the country level to estimate the return on investment over a five-year timeframe. The study concluded that this program generates a global return of $4.28–$11.88 (after investment cost). Gao and co-workers [47] assessed the economic performance of a workplace-delivered intervention to reduce sitting time among desk-based workers. The incremental cost-efficacy ratios ranged from $6.28/minute reduction in workplace sitting time to $8.45/minute reduction in overall sitting time. The intervention was cost-effective over the lifetime of the cohort when scaled up to the national workforce, and provides important evidence for policy-makers and workplaces regarding the allocation of resources to reduce workplace sitting.

## 3. Materials and Methods

### 3.1. Study Design

The present study was carried out from 2011 to 2018, in a workers’ occupational healthcare centre in the oil industry in Bahia, Brazil. It involved the integration of distinct methodological strategies for the development of WHAM, such as the development of a conceptual model, action research, statistical validation, and S-ROI analysis. The study involved two experts who had been working in the field of occupational health for fifteen years, with an emphasis on ergonomics and health management, an interdisciplinary approach, and a database composed of a population group and sample of workers, numbering 1275 and 965 individuals, respectively (Table 1).

### 3.2. Data Analyses

Data analyses were carried out using SPSS version 25 for Windows (IBM Corporation, New York, NY, USA). Diagnostics and intervention prevalence were presented as absolute and relative frequencies. Correlations among modifiable health risk factors and health outcomes were performed through the Kendall rank correlation coefficient. Correlations among health indicators and the interdisciplinary risk coefficients were also performed using the Kendall rank correlation coefficient. Hierarchical multiple regression analysis was used to calculate the independent contributions of occupational medicine interdisciplinary, dentistry interdisciplinary, physical education interdisciplinary, nursing interdisciplinary, and nutrition interdisciplinary risk coefficients, to provide an estimate of incremental variance accounting for the Workers’ Health Risk Index (*WHRI*) [48]. This index had already been published, resulting from the classification of workers into three risk ranges—“low”, “moderate”, and “high”. The Durbin–Watson test was applied to detect the presence of autocorrelation at lag 1 in the residuals (prediction errors), through which the hierarchical multiple regression analysis multicollinearity was verified. To lead the application of the WHAM, the “Guidelines for Implementing the Workers’ Healthcare Assistance Model (WHAM)” were developed, which are presented in the Supplementary Materials (Word S1).

### 3.3. Model Development

The “Workers’ Healthcare Assistance Model” is understood as the organization of the conditions necessary to carry out a person-centred care process, about the method, staff, and instruments. The term “process” used in the context of healthcare makes it possible to identify, understand, describe, explain, and predict the needs of a person, family, or community at a given moment in the health and disease process, demanding professional care by health specialists. Therefore, WHAM presupposes a set of actions, through certain means of action, regulated by a course of thinking; that is, through a conception of workers’ health, WHAM’s origin and its potential to transform itself or to be transformed.

**Figure 1:**
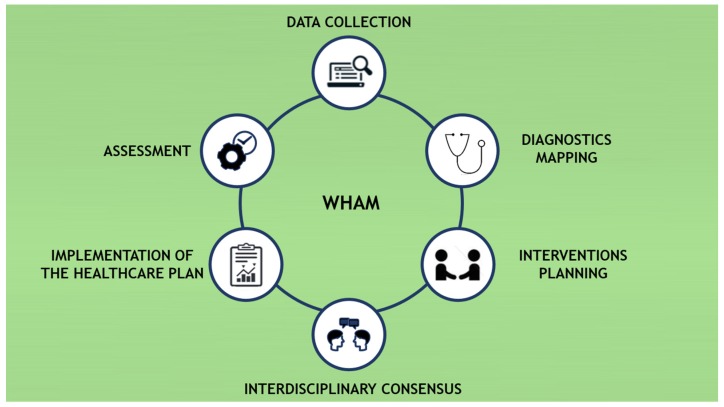
Phases in the Workers’ Healthcare Assistance Model (WHAM).

To compose the WHAM, the Interdisciplinary Workers’ Health Approach Instrument (IWHAI) [49], a tool that had already been published, was used as a data collection instrument, aiming to collect data from 43 health indicators. To map the diagnoses, the health taxonomies were used, while the *WHRI* [48] was used to prioritize the health risks of the workers. Figure 1 shows the main stages of integrating the WHAM.

#### 3.3.1. Data Collection

The data collection stage aimed to identify health problems, as well as the efficient and targeted recording of the workers’ needs in its broadest sense. For this, the IWHAI [49] was chosen. It allows structured data collection, covering the disciplines of medicine, dentistry, nursing, nutrition, and physical education, as well as environmental, occupational, behavioural, personal, and metabolic factors. It is composed of in 5 dimensions with 43 indicators, totalling 215 sub-indexes with closed response coding, where zero represents non-existent or inadequate control of risk and four represents optimal control of risk, arranged in the following scale: 0 = non-existent or inadequate; 1 = tolerable; 2 = reasonable; 3 = good; 4 = excellent.

#### 3.3.2. Diagnostics Mapping

For the diagnostics mapping stage, it was necessary to define taxonomies that encompass the complexity of the workers’ health field, especially those related to the health, environment, and work triad. The following codes were used for medical, dental, nursing, nutritional, and physical education factors: (i) International Classification of Diseases (ICD 11) [50]; International Classification of Nursing Practice (CIPE^®^) [51,52]; International Dietetics and Nutritional Terminology (IDNT) [53]; and the International Classification of Functioning, Disability, and Health (ICF) [54].

#### 3.3.3. Intervention Planning

For the intervention design stage, it was necessary to define classifications that encompass proposals for interventions, which include ecological and occupational care. For each mapped diagnosis, an intervention must be associated. During the attendance of the worker, priority is given to diagnoses for health indicators that are classified as control or health conditions: 0 = non-existent or inadequate; 1 = poor; 2 = reasonable.

#### 3.3.4. Interdisciplinary Consensus

This consists of a discussion amongst the interdisciplinary health team to validate the perceptions [55] raised by professionals in each area during the attendance of workers, sharing the diagnoses and interventions proposed by each discipline. The IWHAI [49] was used as a guiding instrument for data collection. For support of the team decisions regarding the hierarchy of priority interventions, the *WHRI* [48] was used, allowing multidisciplinary (by dimension) and interdisciplinary (association of all dimensions) risk classifications. The classifications comprise three ranges: “low”, “moderate”, and “high”. Since 64% of the sample age is above 40 years and the gender proportion of male to female is very high, the effects of these factors were controlled in this step by the *WHRI* [48] assessment. As the workers’ ages increase, the risk indicator also increases; the same happens for male and female workers for some sex-related diseases, such as the higher susceptibility by men to develop cardiovascular diseases and alcohol abuse. For this reason, when *WHRI* [48] is applied, each worker will have two risk indicators influencing the indicators of health behaviours and outcomes: a risk indicator related to the workers’ age, whereby the older the worker, the higher their risk indicator; and another risk related to their sex, whereby female or male gender will have different impacts on health behaviours and outcomes. The final *WHRI* [48] score is mediated by the workers’ age and sex.

The *WHRI* [48] dimension that has the greatest weight in the interdisciplinary context is designated as the worker case manager (WCM) and will assume technical responsibility concerning care management.

#### 3.3.5. Implementation of the Healthcare Plan

The care plan (CP) is an interdisciplinary document, composed of relevant IWHAI indicators with their respective diagnoses and associated interventions, in addition to the definitions of the implementation and deadline. For the implementation of the CP, the WCM must bring together the interdisciplinary intervention team (IIT), ratify the CP, and proceed with the treatment of the proposed actions through interdisciplinary assistance, group work, and collective and environmental interventions. After validation of the CP by the IIT, the workers are involved in discussing the CP and implementing it at the individual level.

#### 3.3.6. Assessment

The assessment stage deals with the follow-up and monitoring of the workers to the effectiveness of the implemented health interventions. For this, it is necessary to systematically reassess the *WHRI* [48]. The attendance took place in a single period (shift) by each member of the interdisciplinary team, with an average time of 40 min for each consultation and a total time of 3.5 h for each worker in the health service.

### 3.4. WHAM Validation

To validate the WHAM, the data collected in 2018 were used in a representative sample of the population of 965 workers, where attendance by the interdisciplinary team occurred at the same time. Through statistical tests, the intention was to identify the prevalent diagnoses and interventions, how the modifiable factors are related to health outcomes in this sample, and the impact each dimension has on the *WHRI* [48], i.e., if the joint use of these dimensions contributes to greater robustness and explanatory capacity of the WHAM.

### 3.5. Assessment of Sustainable Return on Investment (S-ROI)

To assess the cost-benefit (*CB*) relationship of implementing WHAM, interventions directed at workers with coronary heart disease (CHD) and diabetes in the period ranging from 2011 to 2018 were analyzed. The effectiveness of the intervention was based on the results of epidemiological studies over this period. Brazilian national data were used to estimate the average annual benefits of preventing direct medical costs for diseases.

The analytical tool WELLCAST ROI™ [56], developed to justify the approval of disease prevention and management programs, was used to calculate the S-ROI. For this, the following steps were taken: (i) determine the incidence of the pre-program disease; (ii) determine all costs associated with the disease, either medical costs (for CHD patients, the Framingham model [57] was used to calculate incidence pre and post-program for a period of 10 years, assuming changes in Low-density lipoprotein (LDL) cholesterol, and systolic and diastolic pressure risk factors; for patients with diabetes mellitus, the reduction in the progression of diabetes comorbidities over 10 years was calculated, based on the reduction of glycemia, considering the retinopathy, kidney disease, neuropathy, and microangiopathy comorbidities) or economic costs (monthly salary data, loss of daily productivity, medical inflation rate, among other rates estimated by WELLCAST ROI™); (iii) define the program and its cost; (iv) determine the effectiveness of the program in reducing costs; (v) subtract post-program costs from pre-program costs to determine reductions; and (vi) apply the concepts of net present value (*NPV*), internal rate of return (*IRR*), and *CB* to determine the S-ROI.

### 3.6. Ethical Approval

In all stages of the study, the recommendations and guidelines of Resolution 466/2012 [58] of the Brazilian Ministry of Health on ethical aspects regulating research with human beings, approved by the Research Ethics Committee of the Bahia School of Medicine and Public Health and Certificate of Presentation for Ethical Consideration (CAAE) 84318218.2.0000.5544, were followed. All subjects gave their informed consent for inclusion before participating in the study.

## 4. Results

The prevalent diagnoses and their respective interventions by dimension are presented in detail in Table 2.

In the physical education dimension, the most prevalent diagnosis is “regular aerobic capacity” (76.3 %), with the most prevalent intervention being “encourage thinking about starting a physical activity program, warning about the harm of physical inactivity” (84.8 %). In the field of nursing, the “impaired ability to perform leisure activities” (100.0 %) stands out as the most prevalent diagnosis, followed by the need to “promote ergonomic comfort” (99.0 %) as the most necessary intervention. In the field of medicine, “primary essential hypertension” emerges as the diagnosis with the highest prevalence among workers (87.2 %), preceded by “encourage health-seeking behaviour” (95.5 %) as the intervention with the greatest application within this sample. At the nutritional level, “excessive alcohol intake” is the most prevalent (99.0 %), with the intervention with the greatest application focusing on the need for “adequate macronutrients” (87.6 %). Finally, in the field of dentistry, the most prevalent diagnosis is identified as “other somatoform disorders related to stressful events—bruxism” (97.1 %), with the predominant intervention being “guide to restorative treatment with external dentist” (72.9 %).

Table 3 shows the statistically significant correlations between modifiable health behaviours and health outcomes.

Moderate correlations in Table 3 (*τb* ≥ 0.30) are identified as follows: between diabetes mellitus and altered blood glucose (*τb* = 0.65), energy balance intake (*τb* = 0.48), and the level of food knowledge (*τb* = 0.46); between arterial hypertension and the contemplation stage for physical activity (*τb* = 0.31); between the musculoskeletal pathology and the feeling of pain (*τb* = 0.40); between psychiatric pathology and energy balance intake (*τb* = 0.36); between triglycerides and energy balance intake (*τb* = 0.32); between caries and oral hygiene quality (*τb* = 0.30); between periodontal disease and periodontal condition (*τb* = 0.76), oral hygiene quality (*τb* = 0.58), level of food knowledge (*τb* = 0.31), altered blood glucose (*τb* = 0.45), energy balance intake (*τb* = 0.44), and simple carbohydrate intake (*τb* = 0.33).

The results are shown in Table 4 show which indicators are most correlated with each coefficient of each dimension of interdisciplinary risk.

The values presented in Table 4 make it clear which indicators are most correlated with multidisciplinary risk; the worse an indicator is, the more the risk increases. Thus, in the field of physical education, it appears that the indicator of the contemplation stage for physical activity is the one that is most strongly correlated (*τb* = 0.59). In nursing, the physical aspects of ergonomic risks have the most significant correlation (*τb* = 0.44). In the field of medicine, diabetes mellitus is the most disturbing indicator (*τb* = 0.60). In nutrition, alcohol consumption presents the strongest correlation (*τb* = 0.45). Finally, the highest correlation of all is for oral lesion on soft or hard tissue, which is the most significant indicator in the field of dentistry (*τb* = 0.82).

Hierarchical regression analysis was applied to understand whether the variables or dimensions under analysis explain a statistically significant amount of the variance of the dependent variable to be tested—in this case, the *WHRI* [48] *(*Table 5). A comparison of stages is made by gradually adding each independent variable in each stage, to understand if the combination of the dimensions explains more than considering them separately.

It can be observed that as the dimensions under analysis are added, the model becomes more robust and has greater explanatory capacity for the dependent *WHRI* [48] variable. Thus, when comparing the first stage (step 1) with the last stage (step 5), an increase of 52% in the explained variance of the *WHRI* is observed with the 5 analyzed dimensions, showing values of 34% (*R*^2^ = 0.34) and 86% (*R*^2^ = 0.86), respectively. Medicine is the dimension with the most significant impact on the model (*B* = 0.205; *t* = 35.03; *p* < 0.05) and nursing has the least impact on the model (*B* = 0.168; *t* = 20.76; *p* < 0.05). The model’s final expression is as follows:

(1) $$\begin{array}{l} WHRI=0.035+\left(0.205\times Medicine\right)+\left(0.194\times Nutrition\right)\\ \;+\left(0.179\times Physical\;Education\right)+\left(0.168\times Nursing\right)+(0.166\\ \;\times Dentistry) \end{array}$$

After analyzing the robustness of WHAM, its economic sustainability was assessed using the WELLCAST ROI™ tool. For the analyzed time period and based on the *NPV* of USD 23,363.29/per worker, the *IRR* of 85.5%, and the *CB* of 1.85:1, the S-ROI was determined, suggesting that WHAM is economically sustainable.

## 5. Discussion

Given its complexity, the field of healthcare requires the mobilization of specialists from different areas, with the aim of promoting comprehensive and integrated care for workers. Based on an approach aimed at changing behaviors and adopting healthier lifestyles, going beyond the mere medicalization or treatment of diseases, the interdisciplinary care on which the WHAM model is based resulted in the data presented in Table 2. In view of the most prevalent diagnoses identified for each of the integrated dimensions, an intervention was generated that promotes worker autonomy and the maintenance of healthy lifestyles and behaviors, such as physical activity, healthy eating, non-consumption of alcohol and tobacco, good oral hygiene, balanced social and environmental relations, and decent work habits [55]. At this level, hypertension or diabetes mellitus diagnosis is highlighted, suggesting healthy behaviors or healthier eating habits interventions. As Eng and collaborators [59] state, the workplace is a key space for guidance around healthy behaviors and the reduction of non-communicable diseases (NCDs), such as diabetes mellitus and arterial hypertension. Viterbo and co-authors [23] report that long-term interdisciplinary practice has had very positive and significant effects on reducing NCDs. Hochart and Lang [60] also mention in their study that the implementation of a comprehensive care program in the workplace with the aim of modifying health risk behaviors resulted in a decrease in workers in the high and medium risk ranges and in the maintenance of health for those that were in the low risk range. The same is true for the issue of oral health, a problem that is related to other serious diseases [61,62], and which is solved through the implementation of regular programs for the adoption of oral hygiene behavior among workers, as reported by Viterbo and collaborators [63]. Supporting these results, and in order to reinforce the importance of an integral look at workers’ health, Table 3 presents the results between the behaviors (modifiable factors) and the results for workers’ health. An overview of these results makes the connections between behaviors and health outcomes even more evident, as well as between the results themselves. In this case, an individual look at a worker would not allow one to understand them as a whole, contributing to fragmentation. Certain associations exemplify this idea, namely between the level of food knowledge and the type of food, identified by the energy balance intake, altered blood glucose, and diabetes mellitus. A similar relationship was identified in a review by Sami and co-authors [64], in which guidance towards healthier eating practices reduced the level of diabetes and prevented associated complications. The study by Holynska and colleagues [65] showed that the level of food knowledge is effectively related to nutrient intake, as this study also demonstrated. In line with this, Breen et al. [66] argued that the level of food knowledge enhances the choice of food, thus optimizing the quality of life of people with diabetes.

Table 4 shows the results of the indicators that are most correlated with the risk of each analyzed dimension, making it possible to identify those that contribute most to the increased risk in that dimension. The strongest correlation belongs to the field of dentistry, more specifically for oral lesions increasing the health risk of these workers. According to Warnakulasuriya et al. [67], conducting screening programs using valid visual inspection method to detect potentially malignant oral disorders within a workplace is not only feasible, but also effective. In terms of physical activity, the indicator that has the strongest correlation is that of the contemplation stage for physical activity; that is, the predisposition to start a physical activity. In the review by Jirathananuwat and Pongpirul [68], the 48 studies analyzed demonstrated that the workplace can play an important role in promoting regular physical activity among workers. Ergonomic risks in the workplace are, in this context, assumed to be the most correlated with risk in the field of nursing. This has been documented in several studies, namely by Skovlund et al. [69] and Welch et al. [70]. Since workers spend long hours of their day at the workplace, an additional concern regarding workplace ergonomics must be considered, as correct adaptation will result not only in promoting the well-being of workers, but also in reducing medical costs for employers, as reported by Munir et al. [45], Gao et al. [47], and Welch et al. [70]. In terms of pathologies, diabetes mellitus is the indicator that most contributes to risk in the dimension of medicine. In the reviews by Hafez [71] and Gan [72], the workplace is an important space for effective reduction of diabetes mellitus.

### Implications for Workplace

Some of the results in this study will have a direct implication in the workplace context, thus a more detailed specific analysis is necessary. The results regarding the WHAM robustness (Table 5) make it clear that the combination of technical and scientific knowledge in the work context results in a better understanding of the workers’ global health. This result makes it possible to effectively verify that the interdisciplinary approach translates into gains in health, and that it must be adopted as a matrix in all work contexts, particularly those referring to a higher exposure risk and greater number of employees, as already identified in the studies by Viterbo et al. [23], Clark et al. [73], and Costa et al. [74].

Considering that health promotion and prevention actions can influence the health habits and behaviors of workers, they can also reduce health costs. The literature review [38,75,76,77] suggests that programs based on behavior change theory and using personalized communication and individualized counselling for high-risk individuals are likely to produce a positive return on the amount invested in these programs. The assessment of S-ROI in the specific model under investigation (WHAM) corroborates other studies carried out in the workplace [41,44,45,47], showing positive financial results and reinforcing the advantages of applying WHAM, which in addition to directing investment in health strategies that are proven to be a priority, enables the optimization of financial resources, resulting in an S-ROI of 85.5% for interdisciplinary, integral, and integrated interventions for the community of workers with a high risk level.

## 6. Conclusions

The search for a healthcare model for workers that is oriented towards integrated care, expanded health needs, economic sustainability, and which overcomes the problems arising from the hegemony of the biomedicine paradigm, such as the excessive use of technologies and focus on curative actions of diseases, is one of the great challenges of the Brazilian health system today. This scenario is strongly present in Brazilian scientific production and is reflected in national and international policies through legislation and public initiative.

The results obtained with the practical application of WHAM in the oil industry in Bahia, Brazil, demonstrated the potential of the model, where the articulated and hierarchical management of the various indicators of workers’ health makes it possible to direct practices aimed at the cause and not at the effect or symptom. At the individual level, the model presented an interdisciplinary diagnosis of the health conditions of each worker, correlating the modifiable health factors and their respective impacts. The presentation of information to individuals promoted autonomy and empowered workers to change behaviors that negatively interfere with health conditions. At the collective level, the application of the model demonstrated the correlation between health indicators and interdisciplinary risk in the studied context, encouraging the creation of strategies aimed at the most critical conditions, as well as the design of preventive interventions. The robustness of the model highlights this same potential, in addition to the related optimization of financial resources of 85.5% for interdisciplinary interventions.

The absence of a similar model in occupational health is a limitation of this study since comparative analyses in the context of this work are not possible. The application of WHAM in different healthcare contexts is suggested in future studies, as well as carrying out analyses of the model’s effectiveness by comparing the population’s epidemiological results and studying the S-ROI.

The different theoretical contributions to the theme of this study, as well as the results found, lead to the understanding that WHAM can be considered as a model capable of encompassing the complexity of the field of occupational health, considering the interdisciplinary approach, risk management, and comprehensive and integrated care, in addition to accounting for economic sustainability for companies investing in healthcare.

## Figures and Tables

**Table 1:** Population and sample characterization.

	Population *n* (%)	Sample *n* (%)	Difference (%)	*p*
**Sex**				
Male	1117 (87.6)	884 (91.6)	4.0	>0.05
Female	158 (12.4)	81 (8.4)	−4.0	
**Age Group**			0.7	
≤29	50 (3.9)	44 (4.6)	−0.5	
30 a 39	350 (27.5)	261 (27.0)	2.5	
40 a 49	245 (19.2)	209 (21.7)	−1.1	
50 a 59	556 (43.6)	410 (42.5)	−1.6	
≥60	74 (5.8)	41 (4.2)	4.0	
**Total**	1275	965		

**Table 2:** Diagnosis and intervention prevalence by dimension.

Dimension	Indicator (Assessment Number)	Prevalent Diagnostics	*n* (%)	Prevalent Intervention	*n* (%)
Physical Education	Physical Activity Level (527)	General Physical Resistance—Sedentary	140 (26.6)	Guide and Clarify the Frequency and Duration of Activities Performed to Increase the Level of Physical Activity	290 (55.1)
	Contemplation Stage for Physical Activity Practice (322)	Serious Difficulty in Making Decisions—Contemplation	96 (29.8)	Encourage Thinking about Starting a Physical Activity Program, Warning about the Harm of Physical Inactivity	273 (84.8)
	Feeling of Pain (71)	Moderate Pain	34 (47.9)	Guide to Work Physiotherapy	35 (49.3)
	Cardiorespiratory Fitness (135)	Regular Aerobic Capacity	103 (76.3)	Recommend Specific Physical Activity	89 (66.4)
	Abdominal Strength Level (222)	General Physical Resistance—Regularly Active	58 (26.1)	Stimulate and Guide for Resistance Exercise	134 (60.4)
	Flexibility Level (386)	Mobility of Several Joints—Weak Moderate Disability	85 (22.0)	Encourage and Guide for Flexibility Exercise	273 (70.7)
	Manual Gripping Force (121)	General Physical Resistance—Regularly Active	32 (26.4)	Stimulate and Guide for Resistance Exercise	93 (77.5)
Nursing	Ergonomic Risks—Physical Aspects (193)	Impaired Ergonomic Condition	148 (76.7)	Promote Ergonomic Comfort	191 (99.0)
	Ergonomic Risks—Organizational Aspects (46)	Stress due to Change or Transfer of Environment	16 (34.8)	Obtain Data on Ability to Manage Stress	19 (42.2)
	Work Environment Health Conditions (140)	Impaired Health Surveillance	133 (95.0)	Inspect the Workplace	100 (71.4)
	Family Relationships (25)	Impaired Family Process	9 (36.0)	Support Family Coping Process	12 (48.0)
	Social Aspects—Leisure (14)	Impaired Ability to Perform Leisure Activities	14 (100.0)	Implement Leisure and Fun Activities for Workers and Family Members	7 (50.0)
	Self-Care Level (585)	Health-Seeking Behavior	165 (28.2)	Reinforce Positive Behavior	106 (18.1)
Medicine	Tobacco Use (22)	Tobacco Use	16 (72.7)	Encourage Health-Seeking Behavior	21 (95.5)
	Stress Level and Symptoms (64)	Symptoms and Signs Related to Emotional State	13 (20.3)	Encourage Health-Seeking Behavior	38 (59.4)
	Dyslipidemia (515)	Pure hypercholesterolemia	179 (34.8)	Encourage Health-Seeking Behavior	362 (70.4)
	Diabetes Mellitus (68)	Non-insulin-dependent	53 (77.9)	Guide to Specialist	42 (61.8)
	Systemic Arterial Hypertension (94)	Primary Essential Hypertension	82 (87.2)	Encourage Health-Seeking Behavior	51 (54.3)
	Musculoskeletal Pathology (111)	Low Back Pain	21 (18.9)	Encourage Health-Seeking Behavior	69 (62.2)
	Psychiatric Pathology (10)	Generalized Anxiety	2 (20.0)	Encourage Health-Seeking Behavior	7 (77.8)
	Altered Glycemia (93)	Increased Blood Glucose	62 (66.7)	Guide to Specialist	50 (53.8)
	Altered Blood Pressure (220)	Primary Essential Hypertension	111 (50.5)	Guide to Specialist	96 (43.6)
Nutrition	Energy Balance Intake (339)	Excessive Estimated Energy Intake	239 (70.5)	Adequate Macronutrients	296 (87.6)
	Simple Carbohydrate Intake (148)	Excessive Carbohydrate Intake	74 (50.0)	Adequate Macronutrients	83 (56.5)
	Saturated Lipids Intake (47)	Lipid Type Intake in Disagreement with Needs	30 (63.8)	Adequate Macronutrients	17 (36.2)
	Sodium Mineral Intake (3)	Excessive Oral Intake	2 (66.7)	Instruct Knowledge Related to Nutrition	2 (66.7)
	Fibre Intake (240)	Inadequate Fiber Intake	224 (93.3)	Adequate Macronutrients	92 (38.8)
	Alcohol Consumption (196)	Excessive Alcohol Intake	194 (99.0)	Guide on Alcohol Consumption	147 (75.4)
	Level of Food Knowledge (289)	Limited Adherence to Nutrition Recommendations	48 (16.6)	Promote Continued Food and Nutrition Education	245 (84.8)
	Body Weight Condition (596)	Overweight—Obesity	312 (52.3)	Modify the Distribution, Type, or Amount of Food Nutrients Within Meals or over Time	469 (78.8)
	Altered Triglycerides (268)	Change in Laboratory Values Related to Nutrition	189 (70.5)	Modify the Distribution, Type, or Amount of Food Nutrients Within Meals or over Time	227 (85.0)
Dentistry	Oral Hygiene Quality (803)	Adequate Oral Hygiene	438 (54.5)	Prophylaxis, Topical Application of Fluoride, and Guidance on Correct Oral Hygiene	420 (52.0)
	Periodontal Condition (378)	Supragingival Tartar	223 (59.0)	Supragingival Tartarectomy, Prophylaxis, Topical Application of Fluoride, and Guidance on Brushing Technique and Wire Use	260 (67.0)
	Bruxism (34)	Other Somatoform Disorders Related to Stressful Events—Bruxism	33 (97.1)	Guide to Specialist	13 (35.1)
	Periodontal Disease (27)	Chronic Periodontitis	18 (66.7)	Guide to Periodontist Treatment	17 (60.7)
	Caries (84)	Dentin Caries	50 (59.5)	Guide to Restorative Treatment with External Dentist	62 (72.9)
	Oral Lesion on Soft or Hard Tissue (3)	Leukoplakia and Other Disorders of the Oral Epithelium, Including the Tongue	1 (33.3)	Guide to Specialist	2 (66.7)

**Table 3:** Significant (*p* < 0.05) correlations among modifiable health behaviours and health outcomes.

	Health Outcomes							
Modifiable Health Behaviors	1	2	3	4	5	6	7	8
Altered Blood Glucose	0.65		0.25			0.14		0.45
Stress Level and Symptoms								
Altered Blood Pressure	0.21					0.21	0.18	
Alcohol Consumption		0.09				0.13		
Social Aspects - Leisure								
Self-Care Level		0.08				0.25		
Family Relationships								
Body Weight Conditions		0.06	0.15			0.23		
Energy Balance Intake	0.48	0.07	0.21		0.36	0.32	0.17	0.44
Simple Carbohydrate Intake			0.16			0.25		0.33
Saturated Lipids Intake		0.11						
Sodium Mineral Intake						0.07		
Fibre Intake						0.06		
Tobacco Use								
Level of Food Knowledge	0.46	0.11	0.25			0.28	0.18	0.31
Oral Hygiene Quality			0.14			0.12	0.30	0.58
Cardiorespiratory Fitness						0.08		
Contemplation Stage for Physical Activity			0.31			0.09		
Handgrip Strength			0.15					
Physical Activity Level			0.29			0.10		
Abdominal Strength Level			0.18			0.12		
Feeling of Pain				0.40				
Flexibility Level			0.19			0.12		
Bruxism					1.00	0.27		
Periodontal Condition						0.10	0.26	0.76

Note: 1—Diabetes mellitus; 2—Dyslipidemia; 3—Arterial hypertension; 4—Musculoskeletal pathology; 5—Triglycerides; 6—Caries; 7—Periodontal disease.

**Table 4:** Correlations among health indicators and the interdisciplinary risk coefficients.

Indicators	Multidisciplinary Risk Coefficient				
	Physical Education	Nursing	Medicine	Nutrition	Dentistry
Physical Activity Level	−0.57 *				
Contemplation Stage for Physical Activity Practice	−0.59 *				
Feeling of Pain	−0.31 *				
Cardiorespiratory Fitness	−0.32 *				
Abdominal Strength Level	−0.47 *				
Flexibility Level	−0.41 *				
Manual Gripping Force	−0.22*				
Ergonomic Risks—Physical Aspects		−0.44 *			
Ergonomic Risks—Organizational Aspects		−0.13 *			
Work Environment Health Conditions		−0.26 *			
Family Relationships		−0.16 *			
Social Aspects—Leisure		−0.03			
Self-Care Level		−0.07 *			
Tobacco Use			−0.52 *		
Stress Level and Symptoms			−0.22 *		
Dyslipidemia			−0.39 *		
Diabetes Mellitus			−0.60 *		
Systemic Arterial Hypertension			−0.49 *		
Musculoskeletal Pathology			−0.37 *		
Psychiatric Pathology			−0.28		
Altered Glycemia			−0.25 *		
Altered Blood Pressure			−0.42 *		
Energy Balance Intake				−0.37 *	
Simple Carbohydrate Intake				−0.11 *	
Saturated Lipids Intake				−0.13 *	
Sodium Mineral Intake				−0.04	
Fibre Intake				−0.25 *	
Alcohol Consumption				−0.45 *	
Level of Food Knowledge				−0.18 *	
Body Weight Condition				−0.47 *	
Altered Triglycerides				−0.43 *	
Oral Hygiene Quality					−0.55 *
Periodontal Condition					−0.66 *
Bruxism					−0.34 *
Periodontal Disease					−0.54 *
Caries					−0.37 *
Oral Lesion on Soft or Hard Tissues					−0.82 *

Notes: * significant correlations (*p* < 0.05).

**Table 5:** Hierarchical multiple regression analysis scheme.

Predictor Dimensions	Step 1		Step 2		Step 3		Step 4		Step 5	
	*B*	*t*	*B*	*t*	*B*	*t*	*B*	*t*	*B*	*t*
**Medicine**	0.272	22.28	0.285	24.57	0.234	26.61	0.216	30.91	0.205	35.03
**Dentistry**			0.223	18.66	0.209	21.01	0.195	24.66	0.166	29.35
**Physical Education**					0.179	20.81	0.169	24.93	0.179	29.82
**Nutrition**							0.174	24.06	0.194	29.45
**Nursing**									0.168	20.76
***R***		0.58		0.72		0.82		0.89		0.93
***R*^2^**		0.34		0.52		0.67		0.79		0.86
***R*^2^*_a_***		0.34		0.51		0.66		0.79		0.86

Notes: *B* = unstandardized beta; *t* = *t*-test statistic; *R* = multiple correlation coefficient; *R*^2^ = R Square; R^2^_a_ = Adjusted R Square; R =Step 1: Constant = 0.370, F = 496.6, *p* < 0.001; Step 2: Constant = 0.300, F = 511.7 *p* < 0.001; Step 3: Constant = 0.231, F = 638.5 *p* < 0.001; Step 4: Constant = 0.101, F = 911.1, *p* < 0.001; Step 5: Constant = 0.035, F = 1141.3 *p* < 0.001. Durbin–Watson = 1.506. All predictors are significant at 0.05 level. No multicollinearity was identified.

## Supplementary Materials

The following are available online at https://www.mdpi.com/1660-4601/17/9/3143/s1, Word S1: Guidelines for Implementing the Workers’ Healthcare Assistance Model (WHAM).
