# Supplementary material for: Workers’ Healthcare Assistance Model (WHAM): Development, Validation, and Assessment of Sustainable Return on Investment (S-ROI)
Source: Int J Environ Res Public Health. 2020 Apr 30;17(9):3143. doi: 10.3390/ijerph17093143 (PMC7246570; doi:10.3390/ijerph17093143)
Supplement: Supplementary file 1 [file ijerph-17-03143-s001.pdf]

International Journal of
Environmental Research and Public Health

MDPI

Guidelines For Implementing The Workers' Healthcare Assistance Model (WHAM)

Lilian Monteiro Ferrari Viterbo, André Santana Costa, Diogo Guedes Vidal and Maria Alzira Pimenta Dinis

1. Principles of the Workers' Healthcare Service

1.2. The universality of access to health services at all levels of assistance, including health promotion, disease prevention, and treatment and rehabilitation;

1.3. Comprehensive care understood as an articulated and continuous set of preventive and curative actions and services, both individual and collective, required for each worker at all levels of system complexity;

1.4. Interdisciplinarity is characterized by the intensity of exchanges between specialists and by the degree of real integration of the disciplines for a common purpose, which is the workers' health. This includes health professionals' presuppositions, flexibility, confidence, patience, intuition, adaptability, sensitivity towards other people, risk acceptance, and ability to learn to act in a diverse setting and to accept new roles;

1.5. Person-centred care, where the workers must be treated with dignity, compassion, and respect. Care is coordinated, personalized, and empowering;

1.6. Creation of a bond between the health professional and worker, through friendly human-to-human communication, personalization of needs, and more affective and close relationships;

1.7. Empowerment of workers for self-care, which includes a set of measures that cover technical and ethical guidance for the relationships between individuals and health professionals. The workers must take the leading role in healthcare, just as health professionals must take advantage of all contact opportunities to increase their awareness, activation, and engagement;

1.8. Sharing important information with the workers about their health, involving them in their treatment or complaint, so that they feel that their opinion is considered;

1.9. Search for excellence regarding the patient's experience in the health service, considering humanization, automation of care, and optimization of waiting time, while respecting the feedback of workers and analyzing the "patient's journey";

1.10. Dissemination of information regarding the potential of the health services and their use by workers;

1.11. Information and transparency in healthcare organizations, through the publication of clinical and financial results and indicators of quality, safety, and satisfaction.

2. Workers' Healthcare Service Guidelines

2.1. Develop individual actions for assistance and recovery from injuries, as well as collective actions for promotion, prevention, surveillance of work environments, and processes;

2.2. Intervene in the determinants of workers' health;

2.3. Recognize that interdisciplinarity is a fundamental part of the search for equity and integrality;

2.4. Stimulate intersectoral actions, seeking partnerships that enable the integral development of health actions and changes in organizational culture;

2.5. Strengthen workers' participation as essential in achieving health outcomes, particularly in individual and collective empowerment;

2.6. Encourage health research, assessing the efficiency, efficacy, effectiveness, and safety of the actions provided;

2.7. Disseminate and inform the initiatives developed by health professionals, considering participatory methodologies and the knowledge of the workers and the organization;

3. Skills Required by the Healthcare Professionals

3.1. Interpersonal intelligence is the ability to relate, personally and professionally, with other people, in addition to understanding their desires and thoughts and being able to connect with them;

3.2. Intrapersonal intelligence has to do with the ability to connect with yourself. This means achieving self-knowledge and self-control, thus better-controlling emotions and stress;

3.3. Creative intelligence is the ability to take any of the other intelligence forms and innovatively apply them. This includes being able to escape the pre-defined patterns and responses. Think originally and find innovative solutions to problems;

3.4. Interartificial intelligence is the ability to understand the potential of artificial intelligence and robotics, not only to protect them but also to partner with them. This includes knowing how the potential of new technologies expand the ability to think creatively about solutions and using this to our advantage.

4. Basic Training for Healthcare Professionals

4.1. Epistemological Bases

4.1.1. Dialogical Philosophy

4.1.2. Psychodynamics of Work

4.1.3. Ergology

4.1.4. Activity Clinic

4.1.5. Labor Clinic

4.2. Ethics

4.3. Multi-, Inter-, and Transdisciplinarity

4.4. Patient-Centred Care

4.5. Patient Experience

4.6. Risk Listening

4.7. Injury Minimization

4.8. Humanized Service
